# Introducing the Concept of Consonance-Disconsonance of Best Practice: A Focus on the Development of ‘Student Profiling’

**DOI:** 10.3389/fpsyg.2021.557968

**Published:** 2021-04-30

**Authors:** Huy P. Phan, Bing H. Ngu

**Affiliations:** School of Education, University of New England, Armidale, NSW, Australia

**Keywords:** consonance, disconsonance, optimal best, student profile, optimization, positive psychology, flourishing, motivation

## Abstract

The present study, using a non-experimental approach, investigated a theoretical concept of best practice, which we recently introduced – namely: a ‘state of consonance’ and a ‘state of disconsonance’ of best practice. *Consonance of best practice* posits that different levels of best practice (e.g., low level of best practice *versus* optimal level of best practice), as well as other comparable psychological constructs (e.g., motivation towards learning) would cluster or ‘group’ together. *Disconsonance of best practice*, in contrast, would indicate non-overlapping of contrasting levels of best practice (i.e., low level of best practice *versus* optimal level of best practice). Taiwanese undergraduates (*N* = 831) from five private universities in Taipei City and New Taipei City, Taiwan took part in the study by responding to a suite of Likert-scale questionnaires (e.g., Best Practice Questionnaires, Motivation towards Learning Questionnaire), which took approximately 30–35 min to complete. Cluster analysis, commonly known as *ClA*, was used to analyze the data and seek theoretical understanding into the nature of the consonance of best practice. Results, overall, showed support for our proposition, resulting in four distinct profiles: ‘a Balanced Profile,’ ‘an Intrinsic Motivation Profile,’ ‘a Current Best Practice + Interest Profile,’ and ‘a Current Best Practice + Motivation Profile.’ This evidence, helping to advance further research development, has a number of practical implications for consideration. For example, how could we use the Balanced Profile to develop learning objectives and/or pedagogical practices that would encourage students to enjoy their learning experiences?

## Introducing the Concept of Best Practice: The Importance of ‘Student Profiling’

What is ‘student profiling’ or, alternatively, what does a student’s academic profile actually entail? An academic profile, in a general term, may indicate a *specific pattern in cognition*, *motivation*, *and/or behavior that a student may exhibit* (Phan et al., 2018a). Moreover, an academic profile may reflect a student’s historical background (e.g., his previous failures in mathematics), intellectual curiosity, personal interest, career pathway, and state of engagement or disengagement. For example, within the context of secondary school learning in Physics, say, a student’s ‘motivational profile’ may indicate a number of key characteristics and qualities (e.g., the student’s inclination to go beyond of what is expected of him, academically, in Physics) that would define and reflect his state of motivation. At the same time, according to Phan et al. (2018a), a student’s academic profile may portray his/her expectations, philosophical beliefs, and self-beliefs for learning. From the perspective of education, an academic profile may serve to advise a teacher on the use of appropriate resources and pedagogical practices, which could encourage, foster, and promote an enriching academic profile. From our point of view, we contend that an academic profile may exist on a demarcated spectrum: a positive profile *versus* a negative profile.

In their recent research, Phan et al. (2018a) situated the concept of student academic profile within the context of ‘optimal best practice’ (Fraillon, 2004; Martin, 2006; Phan et al., 2016; Phan et al., 2018b). Specifically, the focus of inquiry delved into a student’s specific pattern of current best practice, as well as his/her optimal best practice in a subject matter. The term ‘best practice,’ according to Ngu et al. (2019), is defined as “a person’s accomplishment of three distinctive areas of personal agency: *acquired knowledge*, *personal experience*, and *personal functioning*.” In terms of academia, for example, the term best practice may relate to a student’s acquired knowledge of Economics 101 (e.g., minimum level of knowledge that he/she would attain), and/or his/her personal enjoyment of Psychology. From this understanding, optimal best practice is therefore concerned with the *maximization* of a person’s acquired knowledge, experience, and/or personal state of flourishing (e.g., feeling good about himself/herself).

In terms of its technical, underlying structure, best practice may differentiate into two distinct levels – namely:

(i) A level of *current best practice*, denoted as L_1_, according to Fraillon (2004) and Phan et al. (2017, 2019a), is defined as a person’s perceived level of functioning at the present time – for example, “what is it that I am capable of at present in Algebra?” (e.g., I am able to solve equations with one unknown, *x*, at present).
(ii) A level of *optimal best practice*, denoted as L_2_, in contrast, is defined as a person’s perceived maximum level of functioning that could be fulfilled and/or accomplished (Fraillon, 2004; Phan et al., 2017, 2019a) – for example, “I perceive and believe that I am capable of accomplishing…. in Algebra” (e.g., I am capable of solving equations with three unknowns, *x*, *y*, and *z*. This accomplishment is my maximum capability).

**FIGURE 1:**
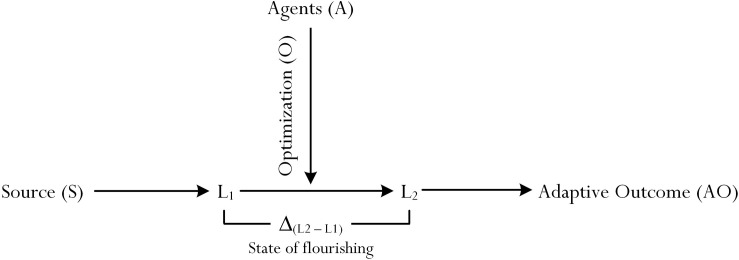
Process of optimization. Source: Phan et al. (2019c). Achieving optimal best practice: An inquiry into its nature and characteristics. *PLOS One* 14(4), e0215732. doi: 10.1371/journal.pone.0215732.

The relationship between L_1_ and L_2_, in its simplistic term, according to Phan et al.’s (2019c) recent study is shown in Figure 1. The uniqueness of Figure 1 lies in the concise representation of the *process of optimization* (Phan et al., 2017, 2019a), which would act to account for a state of flourishing – in this case, defined as a difference between L_1_ and L_2_ [i.e., Δ_(L1 – L2)_]. According to Phan et al. (2019a), the achievement of L_2_ from L_1_ requires some form of optimization, involving the activation and enactment of different types of *educational* (e.g., an appropriate instructional design: Ngu et al., 2018), *psychological* (e.g., belief of personal efficacy: Bandura, 1997), and/or *psychosocial* (e.g., the impact of the home environment: McCartney et al., 2007) agencies. For example, the activation and enactment of self-efficacy (Bandura, 1997) would act to energize specific cognitive processes (e.g., the buoyancy of effort expenditure) and, in turn, helping to optimize a student’s academic learning experience in a subject matter (Phan et al., 2019a, 2020).

Student profiling, from our point of view, may help to explain the L_1_ − L_2_ relationship. From our rationalization and focus of inquiry, as shown in Figure 1, testament of student profiling (i.e., a student’s exhibition of her profile in English composition) may coincide with and complement the theory of optimization (Phan et al., 2017, 2019a) by explaining the interrelatedness between L_1_ and L_2_. The relationship between L_1_ − L_2_ can be explained from the context of academic learning. For example, as shown in Figure 1, a secondary school student’s L_1_ in mathematics learning may consist of her ability to solve equations with one unknown, *x* (e.g., I am capable of solving simple one-unknown equations, for example: *x* + 20 = −4), which then would influence the accomplishment of L_2_ (e.g., the student’s indication in ability to solve equations with two unknowns, *x* and *y*). This theorization contends that, aside of L_1_ being a determinant of L_2_, the quantitative and/or qualitative difference between L_1_ and L_2_, in part, depends on the student’s cognitive level of L_1_ (Phan et al., 2020) – that is, how much does the student know? There are a few empirical research undertakings, which have yielded consistent evidence to support the L_1_ − L_2_ relationship. For example, in a recent study that involved secondary school students, Phan and Ngu (2021) found that L_1_ exerted a positive effect on L_2_ (β = 0.33, *p* < 0.001).

### The Significance of Student Profiling

The study of best practice has substantial daily relevance for students and educators, alike (Phan et al., 2019c, 2020). One notable emphasis for consideration relates to reflective thoughts, articulations, and considered measures, which could help improve a person’s L_2_. To advance this development, we propose an interesting line of inquiry for examination – namely, the extent to which a student’s academic profile could elucidate the relationship between current best practice (L_1_) and optimal best practice (L_2_) in a subject matter. Testament of an academic profile may, in this case, assist in the organization of resources, the design of effective pedagogical practices, and/or the development of policies and/or programs for implementation, in turn facilitating students’ motivational beliefs and learning experiences. To this end, effort has been made recently by Phan et al. (2018a) to study the nature of the concept of academic profile. According to the authors, there are four potential profiles that students may attest and manifest:

(i) The *Exceptional Profile*: ‘High Current Best Practice, High Optimal Best Practice,’ wherein a student reports a high level of current best practice and a high level of optimal best practice. This profile, from our point of view, is healthy, proactive, and motivational.
(ii) The *Realistic Profile*: ‘High Current Best Practice, Low Optimal Best Practice,’ wherein a student reports a high level of current best practice but a low level of optimal best practice. This profile, from our point of view, suggests a student’s conservative sense of self-awareness of his/her capability.
(iii) The *Pessimistic Profile*: ‘Low Current Best Practice, Low Optimal Best Practice,’ wherein a student reports a low level of current best practice and a low level of optimal best practice. This profile, of the four profiles, is pessimistic and negative and may reflect a student’s low level of motivation, helplessness, and uncertainty.
(iv) The *Un-Realistic Profile*: ‘Low Current Best Practice, High Optimal Best Practice,’ wherein a student reports a low level of current best practice and a high level of optimal best practice. This profile, of the four profiles, is positive and optimistic and may reflect a student’s optimism and confidence to succeed in life.

**FIGURE 2:**
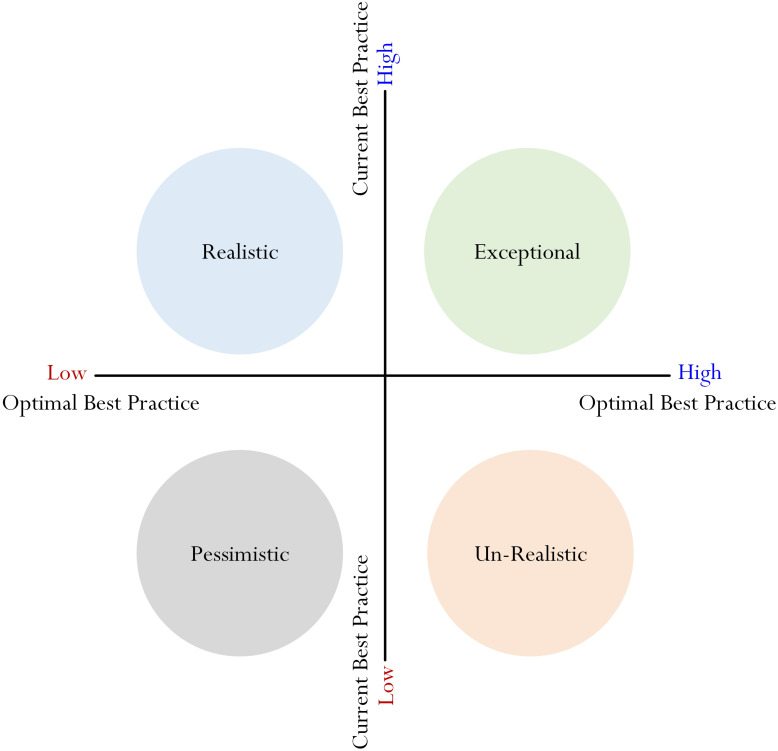
Four profiles of best practice.

The above description, as summarized visually in Figure 2, connotes that each profile would exhibit a set of specific characteristics and qualities. The characteristics of the four profiles, as detailed in Table 1, offer distinct insights into students’ learning and motivational patterns. Moreover, we speculate that educators could use a particular profile (e.g., the Exceptional Profile) as a diagnostic tool to gauge into a student’s learning patterns, motivational beliefs, aspirations, and future outlooks. For example, a student who exhibits the ‘Pessimistic Profile’ may possess a high level of helplessness and a low level of motivation, which would require some form of remediation, personal counseling, etc. A different student, in contrast, may exhibit the ‘Exceptional Profile,’ indicating characteristics of motivation, inspiration, hardworking, etc. Indeed, the uniqueness of ‘academic profiling,’ in accordance with Phan et al.’s (2018a) theorization, lies in its distinct characteristics, helping to identify and discern students’ similarities and differences. From a practical point of view, the use of profiling is advantageous, especially in terms of diagnosis, identification, and the framing of learning objectives and the development of programs and/or policies that could encourage the adoption of the Exceptional Profile.

**TABLE 1:** A summary of different profiles.

Profiles	Current best	Optimal best	Potential characteristics
	practice	practice	
*Exceptional*	High	High	Healthy, proactive, high level of motivation, positive, inspirational, hardworking, high level of confidence, high level of effort
*Realistic*	High	Low	Tempered, self-awareness, confident, hardworking, modest level of confidence, modest level of effort
*Pessimistic*	Low	Low	Pessimistic, low level of motivation, high level of helplessness, uncertainty, negativity, low level of confidence, low level of effort
*Un-Realistic*	Low	High	Optimistic, high level of confidence, low level of effort, unrealistic, ignorance, complacency

### Introducing the Theoretical Concept of Consonance-Disconsonance of Best Practice: Proposition for Consideration

**FIGURE 3:**
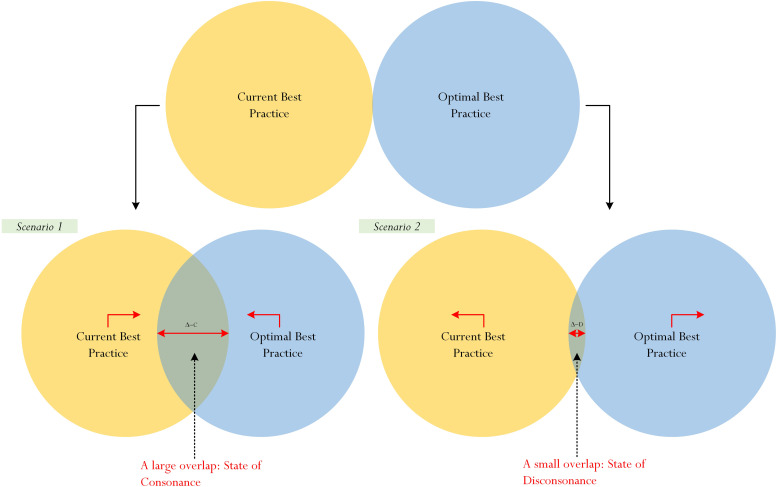
Consonance and disconsonance of best practice. **(A)** Large overlap–indication of consonance. Current and optimal best are moving towards each other. **(B)** Small overlap–indication of disconsonance. Current and optimal best are moving away from each other.

We seek to advance the study of best practice (Fraillon, 2004; Liem et al., 2012; Phan et al., 2016) and, in particular, the inquiry pertaining to the notion of academic profile (Phan et al., 2018a) by focusing on a conceptualization, which we have developed and termed as the ‘consonance and disconsonance of best practice’ (Figure 3). We define the *consonance of best practice* (Figure 3A) as “a ‘closeness’ or the close proximity between a student’s L_1_ and his/her L_2_.” Moreover, referring to our previous discussion, consonance of best practice is similar to the Exceptional Profile (i.e., High Current Best Practice and High Optimal Best Practice) and the Pessimistic Profile (i.e., Low Best Practice and Low Optimal Best Practice) (Phan et al., 2018a). The *disconsonance of best practice* (Figure 3B), in contrast, is defined as “the ‘farness’ between a student’s L_1_ and his/her L_2_.” A state of disconsonance of best practice, in this case, is similar to the Realistic Profile (i.e., High Current Best Practice and Low Optimal Best Practice) and the Un-Realistic Profile (i.e., Low Current Best Practice and High Optimal Best Practice) (Phan et al., 2018a).

Our proposition contends that the dynamic ‘separation’ or ‘movement’ between L_1_ and L_2_ (e.g., compare Figure 3A and Figure 3B) actually distinguishes the consonance of best practice from the disconsonance of best practice and, likewise, the disconsonance of best practice from the consonance of best practice. In accordance with Figure 3A, when both L_1_ and L_2_ are moving towards each other, there would a large overlap, indicating a state of consonance. In relation to Figure 3B, in contrast, when both L_1_ and L_2_ are moving away from each other, there would be a small overlap, indicating a state of disconsonance. This distinction, overall, purports that the two state of best practice (i.e., consonance and disconsonance of best practice) are dynamic and differ in terms of intensity. In the context of schooling, for example, at any moment in time, a student’s level of L_2_ could vary in ‘distance’ from his/her level of L_1_. The distance, or quantitative difference, between L_1_ and L_2_, equating to a state of flourishing (Phan and Ngu, 2017; Phan et al., 2019a), we contend, would reflect a state of consonance of best practice or a state of disconsonance of best practice.

A state of consonance of best practice or a state of disconsonance of best practice is an interesting line inquiry for consideration, especially in terms of educational and non-educational practices for engagement – for example, what educational program could teachers develop, which would encourage a state of consonance of best practice in mathematics learning? By the same token, in terms of sound pedagogical practices, learning objectives, etc., how does an educator determine that a quantitative difference between L_1_ and L_2_ is, in fact, evidence of consonance and not that of disconsonance? Deciding whether a student’s learning experience is one of consonance or disconsonance is subjective and, in part, may depend on his/her *perception of cognitive complexity* (van Merriënboer et al., 2003; van Merriënboer and Sweller, 2005). How difficult is the learning task? Am I able to solve this task? Do I need to put in a lot of effort? Does the task give me mental stress?

We theorize that there is a dynamic movement (i.e., Figure 3A versus Figure 3B) as one progresses from a state of disconsonance to that of consonance, or vice versa. The perceived consonance-disconsonance movement contends that there are two possibilities. The ‘zone of cognitive comfort,’ as possibility 1, depicts that the difference between L_1_ and L_2_ for Scenario 1 (Figure 3A), denoted as Δ-C, is greater than the difference between L_1_ and L_2_ for Scenario 2 (Figure 3B), denoted as Δ-D (i.e., Δ-C > Δ-D). The ‘zone of cognitive discomfort,’ as possibility 2, in contrast, considers that the difference between L_1_ and L_2_ for Scenario 2 is greater than the difference between L_1_ and L_2_ for Scenario 1 (i.e., Δ-D > Δ-C). This theoretical contention posits that a person’s perception of cognitive complexity could, in effect, associate with and/or explain whether there is ‘evidence’ of cognitive comfort or cognitive discomfort. Importantly, from this conceptualization, we argue that evidence of cognitive comfort would result in a student’s report of low mental stress and/or perceived difficulty in his/her learning experiences. Cognitive discomfort, in contrast, would result in a student’s report of high mental stress and/or perceived difficulty in learning experiences.

From the preceding section, we surmise that distinguishing a state of consonance from a state of disconsonance, or vice versa, is insightful as this encouraging feat would help to elucidate theoretical understanding into a person’s perceived cognitive comfort as opposed to that of cognitive discomfort. An inspection of both Figures 3A,B indicates that a shift from cognitive discomfort (i.e., a student’s negative experience) to that of cognitive comfort (i.e., student’s positive experience) would, correspondingly, reflect a shift from a state of disconsonance of best practice to that of a state of consonance of best practice. A shift from cognitive comfort to that of cognitive discomfort, in contrast, would associate with a shift from a state of consonance of best practice to that of a state of disconsonance of best practice. An issue that is of interest for consideration relates, in this case, to comparable psychological and/or educational variables that could closely align with a state of consonance and, by the same token, psychological and/or educational variables that could associate with a state of disconsonance of best practice.

## The Present Study: A Focus on the Consonance of Best Practice

The preceding section, we contend, has established grounding for us to advance further into the study of academic profiling. Our proposed inquiry, in this analysis, considers the potentiality for a state of consonance of best practice to make a meaningful impact on the teaching and learning processes. As a recap, a state of consonance considers a close proximity between L_1_ and L_2_. We propose, however, that aside from this closeness (i.e., a state of consonance), both L_1_ and L_2_ may also associate with other psychological processes and/or outcomes that have similar attributes and characteristics. For example, it is plausible to consider a state of consonance of best practice and a state of positive emotions (e.g., happiness) as being comparable and/or interrelated. Previous research inquiries have, likewise, yielded evidence that illustrates in this instance a ‘consonance’ between anxiety and low academic performance (Pajares and Kranzler, 1995; Pajares and Johnson, 1996; Segool et al., 2013). It is evident, by contrast, that we would not expect to find a state of consonance between a student’s junk food eating habits and his/her engagement of mastery in mathematics learning.

In the context of the present study, we postulate three psychological variables that could closely associate with the consonance of best practice: (i) *motivation towards learning* (Van Damme et al., 2002; Van Landeghem et al., 2002; Van De Gaer et al., 2007, 2009a), (ii) *personal interest in learning* (De Fraine et al., 2005; Van De Gaer et al., 2007, 2009b; Belfi et al., 2012), and (iii) *positive emotions* (Fredrickson, 2000; Tugade and Fredrickson, 2007; Villavicencio and Bernardo, 2013, 2016). It is acknowledged that motivation towards learning, personal interest in learning, and positive emotions (e.g., happiness) are positive in terms of their characteristics, and that they may predict, improve, and/or enhance learning experiences and performance outcomes. Over the past few years, our research interest has led to a number of undertakings, which focused on the study of psychological variables that could explain and predict the achievement of optimal best (Phan et al., 2019c; Phan and Ngu, 2020, 2021). For example, non-experimentally via means of correlational analyses, we note that motivation towards learning positively influences both L_1_ and L_2_.

Other motivational research, likewise, has yielded consistent findings, which emphasize the interrelatedness between comparable variables. In one of the earlier studies that used latent growth modeling (LGM) techniques (McArdle and Nesselroade, 2003; Bollen and Curran, 2006), for example, Van De Gaer et al. (2009a) found a positive association between motivation towards learning and academic self-concept. This inquiry is somewhat different from the study of optimal best practice, but it does provide some empirical insights into the operational nature of motivation towards learning. In a similar vein, other studies have noted the interrelatedness between positive emotions, personal interest, and other related motivational constructs (Tugade and Fredrickson, 2007; Zhu et al., 2009; Villavicencio and Bernardo, 2013, 2016; Phan et al., 2019b). What is of relevance, from our point of view, is that existing evidence supports our conceptualization, which connotes an ‘overlap’ between L_1_, L_2_, motivation towards learning, personal interest in learning tasks, and positive emotions.

**FIGURE 4:**
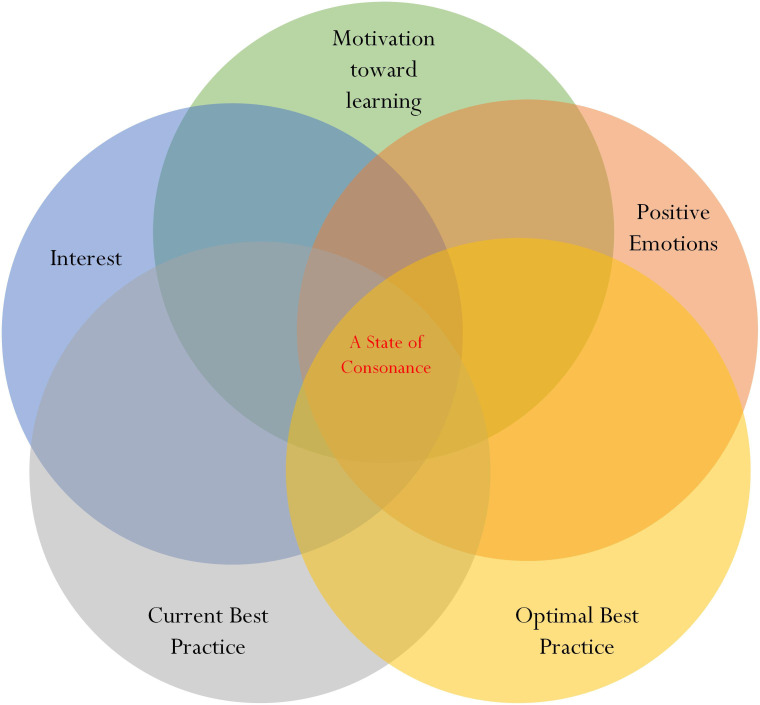
Conceptualization of consonance for investigation.

An ‘overlap’ between comparable educational (e.g., L_1_ and L_2_) and psychological (e.g., motivation towards learning and personal interest in learning) variables, shown in Figure 4, is labeled as ‘A State of Consonance.’ A state of consonance is positive and posits that comparable variables are ‘in tune’ with each other in terms of characteristics, qualities, and understanding. For example, within the context of schooling and academic learning, we would expect to find shared commonalities between a student’s level of L_2_ and his/her indication of motivation towards learning, positive emotions, and/or personal interest in learning. In a similar vein, of course, we would to find a non-overlapping or, alternatively, a state of disconsonance between the student’s level of L_2_ and his/her anxiety.

### Conceptualization of Study

Our conceptualization, overall, is innovative for its proposed investigation into the issue of overlapping and non-overlapping of comparable educational and psychological variables: L_1_, L_2_, motivation towards learning (denoted as MTL), personal interest in learning (denoted as PIL), and positive emotions (denoted as PE). This inquiry may yield a number of valuable outcomes, educationally and non-educationally, and may advance theoretical understanding into the nature of profiling. A state of consonance, for example, may reflect a positive profile and serve as an ideal profile for promotion and cultivation. A state of disconsonance, in contrast, could act as a diagnostic tool, helping educators to develop different preventive measures for the purpose of improvement. At the same time, identified state of consonance and state of disconsonance are insightful, offering potential information into a student’s ‘cognitive profile’ in a subject matter.

In summary, from our conceptualization, we propose that a particular ‘grouping’ would indicate comparable levels of L_1_, L_2_, MTL, PIL, and PE. With reference to the four main profiles that have been cited (Phan et al., 2018a) (e.g., Figure 2), we contend that the Exceptional Profile would indicate some form of overlapping between L_1_, L_2_, MTL, PIL, and PE. In this sense, for the Exceptional Profile, we would expect to find comparable high levels of L_1_, L_2_, MTL, PIL, and PE. By the same token, however, we also expect to find some form of overlapping, but rather relatively low comparable levels of L_1_, L_2_, MTL, PIL, and PE for the Pessimistic Profile. On this basis, as a point of comparison, we could consider two different possibilities. Firstly, a student who is motivated is more likely to be confident, resulting in his/her indication of high L_1_ and L_2_ in a subject matter. At the same time, the motivated student is more inclined to exhibit a corresponding high level of interest in learning and positive emotions because of his/her state of confidence and accomplishment (e.g., the student’s conveyed message of situational and/or dispositional happiness). Secondly, a student who lacks motivation towards learning may indicate low L_1_ and L_2_ in a subject matter. A low level of motivation, in this case, is likely to associate with a low level of interest. At the same time, of course, we consider a student who exhibits a low state of functioning (e.g., L_1_ and L_2_) and motivation to also express an analogous level of negative emotions (e.g., anxiety).

The present study may also yield a number of benefits for institutions, organizations, government officials, etc. One notable benefit, for example, relates to information gathering and data recording for the purpose of policy development, allocation of financial resources, and design and structure of subject contents, courses, and degree programs. Knowing about individual learning and motivational profiles, interestingly, may help an institution’s framing of its entry requirements into different programs. In a similar vein, an institution may provide initial, or foundation, support, which could assist first-year students with their academic adjustment. Finally, we contend, our research inquiry is noteworthy for its empirical contribution and advanced theoretical understanding of academic profiling.

## Materials and Methods

### Sample and Procedure

Eight hundred and forty-eight university students from five private universities in Taiwan (*N* = 527 women, 321 men) took part in the present study. The study reported in this manuscript was approved by our university’s Research Ethics Committee, Approved Number: HE13-025. Educational research in university and school settings is somewhat difficult to undertake nowadays, consequently as a result of time constraint, logistic and resource limitations, institution’s unwillingness to take part (for various reasons), etc. Many researchers would concur that it is a ‘blessing’ nowadays to have a school or a university agreed to take part in an experimental or a non-experimental project. Even still, with consented participation, a 35–40 min duration is manageable but anything longer would impose and pose difficulties. The data collected for this study, somewhat limited, were convenient in terms of sampling – that is, we know of Taiwanese scholars, who are also colleagues, who in turn knew other colleagues who could and were willing to assist with data collection. Ideally of course, with more than 150 colleges and universities available in Taiwan, we would have preferred to have a random sampling (Cohen et al., 2003; Babbie, 2014), which then could offer a more robust representation of the general population.

The dataset was collected during the last week of October, 2018, which took approximately 2 weeks to complete (i.e., last week in October with two of the five universities, and first week in November with the other three universities). The participants were briefed early in October, 2018 that a study was undertaken and that a ‘survey’ would be administered in late October. Coordination of the data collection process across the five universities, located in Taipei City and New Taipei City, was assisted by a Taiwanese colleague who also, at the time of this article, worked in one of the universities.

We chose to use the traditional face-to-face, hard-copy methodological approach rather than an online approach, given that the former would ensure a better response rate. Likert-scale measures, which took approximately 30–35 min to complete, were administered by volunteered lecturers in both lectures and tutorial classes. Participants were given 5 min at the end of the data collection process to ask questions, seek clarification, etc. A few postgraduate students, likewise, assisted in the data entry of the Likert-scale responses, using Excel databases. The Excel databases were eventually merged into one Excel database for statistical analyses, which we described next. A frequency analysis showed 12 different academic subjects that students enrolled in: Pure Mathematics (62 students), Mindfulness: A Focus on Breath (62 students), Chinese (Mandarin) Language (188 students), Mindfulness: A Focus on Zen Philosophy (64 students), Mindfulness: Practice of Enlightenment (38 students), Industrial Engineering (121 students), English Literature (78 students), Financial Mathematics (74 students), Movie Critique (48 students), Chinese Philosophy (21 students), Reflection (15 students), and Essay Writing (60 students).

Finally, we verbally sought permission and informed any participant who did not wish to participate to let us know at the onset. This method of verbally seeking participatory consent without formal written approval from legal guardians and/or parents was logistically convenient and appropriate given the ages of the participants. We also followed our university’s protocols and informed participants at the onset of administration of the Likert-scale questionnaires that participation was voluntary, and that their responses were confidential and only seen for the purpose of data analyses. Aside from voluntary participation and anonymity, no incentive was given to any participant for his/her engagement. With the affordability of time, we asked the participants to spend time to reflect and to consider their responses to the posed questions, and to ask questions for clarification if required.

### Instruments

We adapted three Likert-scale questionnaires (Rating: 1 [Always False] to 5 [Always True]) for usage with Taiwanese university students. The original version of the questionnaires, in English (E), was translated to Chinese Mandarin (CM) using a three-step approach, which existing research has discussed elsewhere: (i) Step 1 involved the translation of the original questionnaires from English to Chinese Mandarin (i.e., E → CM), (ii) Step 2 involved the translation of the translated questionnaires in Chinese Mandarin back to English (i.e., CM → E), and (iii) Step 3 involved comparison of the original English version of the questionnaires (i.e., Step 1) with the translated version of the questionnaires (i.e., Step 2).

Motivational research has emphasized and stipulated the importance of *contextualization* and *specificity* of individuals’ responses (Pajares, 1996; Bandura, 1997). This focus contends that questionnaires posed for answering are meaningful only when their contents are situated within specific contexts – for example, “I have confidence in my ability to do well…” is a general statement that has less predictive power than a more specific statement such as this – “I have confidence in my ability to do well at university in the subjects that I study.” For this study, we focused on five major aspects:

(i) *Current Best Practice*, L_1_, with eight items (Phan et al., 2016), for example: “I am content with what I have accomplished so far for my academic subjects at university” and “I can academically achieve what is being asked of me at university.” The reliability estimate for this subscale is 0.73. A one-factor CFA analysis of the L_1_ subscale (Bollen, 1989; Kline, 2011) showed a moderate goodness-of-fit model [e.g., CFI = 0.98, TLI = 0.95, RMSEA 0.087 (Lo90 = 0.059, Hi90 = 0.118), *p* < 0.05, SRMR = 0.025] with factor loadings from the items to the single latent factor ranging from 0.52 to 0.77 (*Mn* = 0.66, *SD* = 0.09).
(ii) *Optimal Best Practice*, L_2_, with eight items (Phan et al., 2016), for example: “I can achieve much more for the different subjects than what I have indicated through my work so far” and “I want to learn and do more at university.” The reliability estimate for this subscale is 0.74. A one-factor CFA analysis of the L_2_ subscale (Bollen, 1989; Kline, 2011) showed a sound goodness-of-fit model [e.g., CFI = 0.99, TLI = 0.97, RMSEA 0.067 (Lo90 = 0.035, Hi90 = 0.104), *p* > 0.05, SRMR = 0.016] with factor loadings from the items to the single latent factor ranging from 0.51 to 0.75 (*Mn* = 0.62, *SD* = 0.09).
(iii) *Personal Interest in Learning Tasks*, PIL, with eight items (Van Damme et al., 2002), for example: “I really enjoy learning the different academic subjects at university” and “I believe many things we learn at university are not important at all” (−ve item). The reliability estimate for this subscale is 0.89. A one-factor CFA analysis of the PIL subscale (Bollen, 1989; Kline, 2011) showed a sound goodness-of-fit model [e.g., CFI = 0.99, TLI = 0.97, RMSEA 0.059 (Lo90 = 0.044, Hi90 = 0.076), *p* > 0.05, SRMR = 0.022] with factor loadings from the items to the single latent factor ranging from 0.59 to 0.77 (*Mn* = 0.68, *SD* = 0.07).
(iv) *Motivation towards Learning*, MTL, with five items (Van Damme et al., 2002), for example: “I really work hard for all academic subjects at university to get good results” and “There are few academic subjects at university for which I really do my best” (−ve item). The reliability estimate for this subscale is 0.79. A one-factor CFA analysis of the MTL subscale (Bollen, 1989; Kline, 2011) showed a sound goodness-of-fit model [e.g., CFI = 0.99, TLI = 0.97, RMSEA 0.066 (Lo90 = 0.034, Hi90 = 0.103), *p* > 0.05, SRMR = 0.016] with factor loadings from the items to the single latent factor ranging from 0.55 to 0.83 (*Mn* = 0.69, *SD* = 0.11).
(v) *Positive emotions*, PE, with five items, for example: “I am always happy at university” and “I often feel negative with life at university.” The reliability estimate for this subscale is 0.73. A one-factor CFA analysis of the PE subscale (Bollen, 1989; Kline, 2011) showed a moderate goodness-of-fit model [e.g., CFI = 0.98, TLI = 0.92, RMSEA 0.10 (Lo90 = 0.062, Hi90 = 0.144), *p* < 0.05, SRMR = 0.027] with factor loadings from the items to the single latent factor ranging from 0.50 to 0.93 (*Mn* = 0.60, *SD* = 0.18).

Psychometric properties (e.g., factorial validity and reliability estimates) for the five mentioned subscales have been explored in detail and reported elsewhere (e.g., see the following: Van Damme et al., 2002; Van De Gaer et al., 2007; Phan et al., 2019b, c). Our recent research undertakings using structural equation modeling (SEM) techniques (Schumacker and Lomax, 2004; Kline, 2011) yielded consistent reliability estimates for some of the mentioned subscales (e.g., L_1_ and L_2_), and that items loaded onto respective latent factors (e.g., Phan et al., 2019c; Phan and Ngu, 2020, 2021). For example, in a study that involved secondary school students, we found sound factorial structures for the two subscales of best practice (e.g., 0.81–0.95 for L_1_ and 0.75–0.93 for L_2_) (Phan and Ngu, 2021). In an earlier study that focused on university students, we observed similar factorial structures, wherein items loaded onto the two respective latent factors (i.e., 0.54–0.76 for L_1_ and 0.50–0.74 for L_2_) (Phan et al., 2019c).

## Data Analysis

We used SPSS 25 to assist us with our data analysis. There are different models of cluster, for example: connectivity models (e.g., hierarchical clustering), centroids models (e.g., *k*-means algorithm), and distribution models (e.g., expectation-maximization algorithm). Cluster analysis, commonly known as *ClA* and introduced in Tryon (1939), is a popular statistical technique that has often been used in educational and psychological research (e.g., Shavelson, 1979; Egan, 1984; Meece and Holt, 1993; Hwang et al., 2019). One notable aspect of ClA is it enables researchers to locate clusters within a set of responses that have a tendency to be homogeneous. From this understanding, it is likely that we would find high homogeneity within each group (i.e., intra-cluster), and high heterogeneity between two or so groups (i.e., inter-clusters).

The *K*-means algorithm enables educators and researchers to simplify large datasets into smaller and simple datasets (Likas et al., 2003; Jain, 2010; Li and Wu, 2012). The *K*-means algorithm is appropriate for the present study as it provides a statistical basis to help us ‘group’ distinct patterns of student responses into specific groups (e.g., four profiles of levels of best practice, MTL, PIL, and PE). The *K*-means algorithm, as Jain (2010) describes, is still popular despite its introduction more than 50 years ago (Ball and Hall, 1965; MacQueen, 1967; Bock, 2007). In particular, when compared to other algorithms, the *K*-means algorithm is effective for its “ease implementation, simplicity, efficiency, and empirical success” (Jain, 2010). For the purpose of simplicity and not to compound difficulties in terms of readability, we have not delved into the complexity of the *K*-means algorithm approach – we recommend readers to consider some theoretical overviews such as those from MacQueen (1967), Jain (2010), and Li and Wu (2012).

### *K*-Means Cluster Analysis

Before proceeding onto with the formal cluster analysis, we performed an initial data screening analysis to identify for unusual kurtosis and skewness values, extreme outliers for deletion, and missing data responses. At the same time, we conducted a frequency test to ensure that all responses were within the expected range – for example: 1–5. There was no error in terms of incorrect data entry (e.g., the entry of ‘55’ instead of 5 for a response). We noted that this initial data screening test (e.g., stem-and-leaf plots and boxplots) indicated two extreme outliers, which we subsequently deleted. The Mahalanobis distance exceeded the critical χ^2^ for *df* = 3, *p* < 0.001 of 20.25 for 11 cases for deletion. The final sample that we used for our subsequent analyses consisted of 831 students (*N* = 519 women, 312 men).

One limitation of the *K*-means algorithm is that a researcher has to specify the number of clusters at the onset of the analysis. Jain and Dubes (1988), in particular, have noted that the main steps of *K*-means algorithm entail the following: (i) select an initial partition with *K* clusters, (ii) generate a new partition by assignment each pattern to its closest cluster center, and (iii) compute new cluster centers. Steps 2 and 3 are repeated until cluster membership stabilizes (Jain, 2010). From SPSS, we started off with two clusters (i.e., we considered students, in general, to cluster into two opposite groupings: the ‘High L_1_, High L_2_, and high levels of MTL, PIL, and PE’ students *versus* the ‘Low L_1_, Low L_2_, and low levels of MTL, PIL, and PE’ students) by which we then progressed onto different clusters to include eight clusters (i.e., we considered students, in general, to cluster into eight groupings: the ‘High L_1_, High L_2_, and high levels of MTL, PIL, and PE’ students, the ‘Low L_1_, Low L_2_, and high levels of MTL, PIL, and PE’ students, the ‘High L_1_, Low L_2_, and high levels of MTL, PIL, and PE’ students, the ‘Low L_1_, High L_2_, and high levels of MTL, PIL, and PE’ students, the ‘High L_1_, High L_2_, and low levels of MTL, PIL, and PE’ students, the ‘Low L_1_, Low L_2_, and low levels of MTL, PIL, and PE’ students, the ‘High L_1_, Low L_2_, and low levels of MTL, PIL, and PE’ students, and the ‘Low L_1_, High L_2_, and low levels of MTL, PIL, and PE’ students).

The main question then, of course, is related to which cluster (e.g., two clusters *versus* eight clusters) is optimal for discussion purposes. On first inspection, for example, we noted that all seven cluster models were ‘appropriate’ for consideration – in this case, the ANOVA results showed statistically significance for the five constructs under examination. Table 2 shows the ANOVA test result for the two-cluster analysis, whereas the result for the eight-cluster analysis is presented in Table 3. A two-cluster model, we contend, is relatively simple in terms of its profile –the two clusters, for example, differed in scores between 3.15 – 3.46 (Cluster 1: 471 cases) and 3.58 – 4.07 (Cluster 2: 360 cases).

**TABLE 2:** ANOVA results for two clusters.

	Cluster		Error		*F*	Sig.
	Mean square	df	Mean square	df		
L_1_	76.318	1	0.161	829	472.579	0.000
L_2_	73.077	1	0.144	829	508.795	0.000
Motivation towards learning	89.316	1	0.135	829	662.754	0.000
Interest in learning tasks	91.214	1	0.167	829	546.825	0.000
Positive emotions	30.831	1	0.149	829	206.730	0.000

**TABLE 3:** ANOVA for eight-cluster.

	Cluster		Error		*F*	Sig.
	Mean square	df	Mean square	df		
L_1_	20.555	7	0.081	823	255.119	0.000
L_2_	13.920	7	0.115	823	120.960	0.000
Motivation towards learning	20.530	7	0.070	823	294.742	0.000
Interest in learning tasks	23.439	7	0.079	823	294.872	0.000
Positive emotions	9.493	7	0.107	823	88.767	0.000

An eight-cluster model, in contrast, is extremely complex despite our previous proposition. In this case, the eight clusters ranged in scores from 2.86 – 2.96 (Cluster 3: 84 cases) to 3.87 – 4.54 (Cluster 4: 57 cases). *Post hoc* tests using the Bonferroni correction, however, showed that some differences for the five variables between the eight clusters were not statistically significant – for example, the L_1_ score in Cluster 2 and the L_1_ score in Cluster 6 (Δ_Mn_ = 0.064, *p* > 0.05). By randomization, we performed a five-cluster model and, likewise, the *post hoc* tests using the Bonferroni correction produced a few non-statistically significance (e.g., the PIL scores in Cluster 1 and in Cluster 5, Δ_Mn_ = 0.063, *p* > 0.05).

**FIGURE 5:**
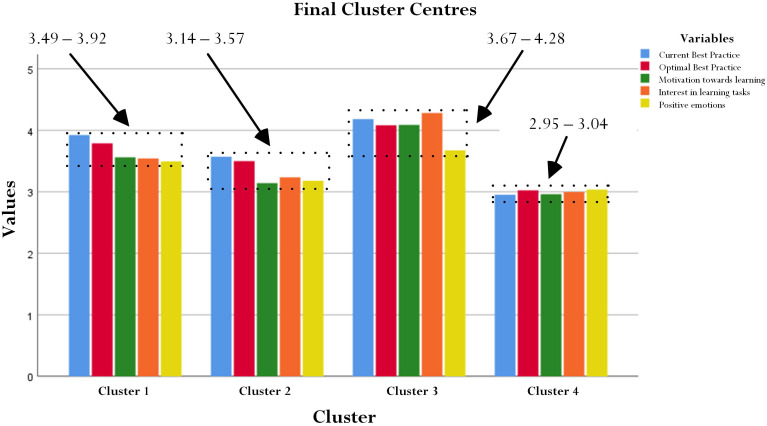
Four-cluster model.

**FIGURE 6:**
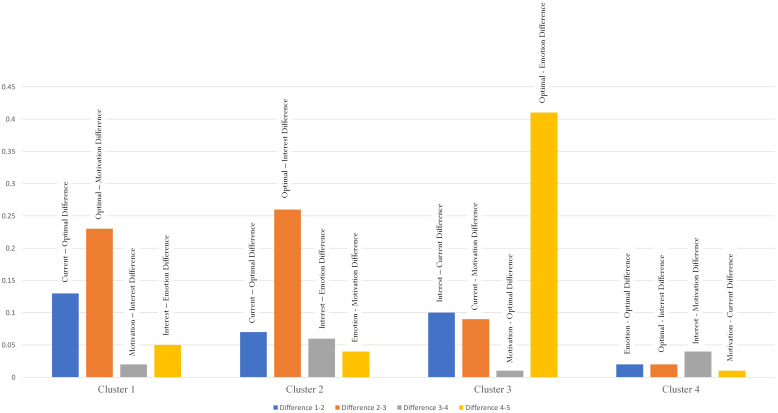
Mean differences between variables for clusters.

A four-cluster model, drawing the limitation of the five-cluster model, was performed and the results for ANOVA are shown in Table 4. In this case, as shown in Figure 5, the four clusters are quite interesting in terms of their scores for the five variables. *Post hoc* tests using the Bonferroni correction, likewise, showed statistically significance (*p* < 0.01 and *p* < 0.001) for the five variables between the four clusters (Table 5). From Figure 5, let us provide a detailed summary of the results in an ascending order: (i) Cluster 4 (119 cases) with scores ranging from 2.95 to 3.04, (ii) Cluster 2 (262 cases) with scores ranging from 3.14 to 3.57, (iii) Cluster 1 (294 cases) with scores ranging from 3.49 to 3.92, and (iv) Cluster 3 (156 cases) with scores ranging from 3.67 to 4.28. The scores of the five variables in each cluster are presented in Table 6. To assist us in our formulation of four distinctive profiles, we computed the difference between two adjacent variables for each cluster – for example, from Table 6, consider Cluster 1 and its respective scores: L_1_ = 3.92, L_2_ = 3.79, MTL = 3.56, PIL = 3.54, and PE = 3.49. On this basis, differences included: L_1_ – L_2_ = 0.13, MTL – L_2_ = 0.23, PIL – MTL = 0.02, and PE – PIL = 0.05. The results for this computation are shown visually in Figure 6.

**TABLE 4:** ANOVA results for four clusters.

	Cluster		Error		*F*	Sig.
	Mean square	df	Mean square	df		
L_1_	40.447	3	0.107	827	376.462	0.000
L_2_	29.206	3	0.126	827	231.071	0.000
Motivation towards learning	40.226	3	0.097	827	413.976	0.000
Interest in learning tasks	47.994	3	0.103	827	464.143	0.000
Positive emotions	13.906	3	0.136	827	102.000	0.000

**TABLE 5:** Post-hoc tests.

Multiple comparisons							
Dependent variable			Mean difference (I-J)	Std. error	Sig.	95% confidence interval	
						Lower bound	Upper bound
L_1_	1	2	0.352*	0.028	0.000	0.278	0.426
		3	−0.258*	0.032	0.000	−0.344	−0.172
		4	0.971*	0.036	0.000	0.877	1.066
	2	1	−0.352*	0.028	0.000	−0.426	−0.278
		3	−0.610*	0.033	0.000	−0.698	−0.522
		4	0.619*	0.036	0.000	0.524	0.715
	3	1	0.258*	0.032	0.000	0.172	0.344
		2	0.610*	0.033	0.000	0.522	0.698
		4	1.229*	0.040	0.000	1.124	1.335
	4	1	−0.971*	0.036	0.000	−1.066	−0.877
		2	−0.619*	0.036	0.000	−0.715	−0.524
		3	−1.229*	0.040	0.000	−1.335	−1.124
L_2_	1	2	0.289*	0.030	0.000	0.209	0.369
		3	−0.294*	0.035	0.000	−0.387	−0.201
		4	0.766*	0.039	0.000	0.664	0.868
	2	1	−0.289*	0.030	0.000	−0.369	−0.209
		3	−0.583*	0.036	0.000	−0.678	−0.488
		4	0.477*	0.039	0.000	0.374	0.581
	3	1	0.294*	0.035	0.000	0.201	0.387
		2	0.583*	0.036	0.000	0.488	0.678
		4	1.060*	0.043	0.000	0.946	1.175
	4	1	−0.766*	0.039	0.000	−0.868	−0.664
		2	−0.477*	0.039	0.000	−0.581	−0.374
		3	−1.060*	0.043	0.000	−1.175	−0.946
Motivation towards learning	1	2	0.420*	0.026	0.000	0.350	0.490
		3	−0.528*	0.031	0.000	−0.610	−0.446
		4	0.600*	0.034	0.000	0.510	0.690
	2	1	−0.420*	0.026	0.000	−0.490	−0.350
		3	−0.948*	0.032	0.000	−1.031	−0.864
		4	0.180*	0.034	0.000	0.089	0.271
	3	1	0.528*	0.031	0.000	0.446	0.610
		2	0.948*	0.032	0.000	0.864	1.031
		4	1.128*	0.038	0.000	1.028	1.228
	4	1	−0.600*	0.034	0.000	−0.690	−0.510
		2	−0.180*	0.034	0.000	−0.271	−0.089
		3	−1.128*	0.038	0.000	−1.228	−1.028
Interest in learning tasks	1	2	0.308*	0.027	0.000	0.235	0.380
		3	−0.738*	0.032	0.000	−0.822	−0.653
		4	0.545*	0.035	0.000	0.453	0.638
	2	1	−0.308*	0.027	0.000	−0.380	−0.235
		3	−1.045*	0.033	0.000	−1.131	−0.959
		4	0.238*	0.036	0.000	0.144	0.332
	3	1	0.738*	0.032	0.000	0.653	0.822
		2	1.045*	0.033	0.000	0.959	1.131
		4	1.283*	0.039	0.000	1.180	1.387
	4	1	−0.545*	0.035	0.000	−0.638	−0.453
		2	−0.238*	0.036	0.000	−0.332	−0.144
		3	−1.283*	0.039	0.000	−1.387	−1.180
Positive emotions	1	2	0.315*	0.031	0.000	0.232	0.398
		3	−0.180*	0.037	0.000	−0.277	−0.084
		4	0.458*	0.040	0.000	0.352	0.564
	2	1	−0.315*	0.031	0.000	−0.398	−0.232
		3	−0.495*	0.037	0.000	−0.594	−0.396
		4	0.143*	0.041	0.003	0.035	0.251
	3	1	0.180*	0.037	0.000	0.084	0.277
		2	0.495*	0.037	0.000	0.396	0.594
		4	0.638*	0.045	0.000	0.519	0.757
	4	1	−0.458*	0.040	0.000	−0.564	−0.352
		2	−0.143*	0.041	0.003	−0.251	−0.035
		3	−0.638*	0.045	0.000	−0.757	−0.519

*The mean difference is significant at the 0.05 level.

**TABLE 6:** Summary of results.

Cluster			
Current Best + motivation (Cluster 1)	Current best + interest (Cluster 2)	Intrinsic motivation (Cluster 3)	Balanced (Cluster 4)
(1) L_1_ (score = 3.92)	(1) L_1_ (score = 3.57)	(1) Personal interest (score = 4.28)	(1) Positive emotion (score = 3.04)
(2) L_2_ (score = 3.79)	(2) L_2_ (score = 3.50)	(2) L_1_ (score = 4.18)	(2) L_2_ (score = 3.02)
(3) Motivation (score = 3.56)	(3) Personal interest (score = 3.24)	(3) Motivation (score = 4.09)	(3) Personal interest (score = 3.00)
(4) Personal interest (score = 3.54)	(4) Positive emotion (score = 3.18)	(4) L_2_ (score = 4.08)	(4) Motivation (score = 2.96)
(5) Positive emotion (score = 3.49)	(5) Motivation (score = 3.14)	(5) Positive eotion (score = 3.67)	(5) L_1_ (score = 2.95)

The solution shown in Table 6 and in Figures 5, 6 is interesting for its potential distinction of four comparable clusters. How do we derive and formulate different clusters given that the results, in general, exhibit similar patterns? Inspecting the results, we note the following clusters:

#### Cluster 4 = ‘A Balanced Profile’

Students for this profile express positive emotions and are quite optimal in their level of best practice. Overall, however, there is a balance in the five variables whereby they do not differ that much in their ‘score differences.’ This profile reflects a measured judgment in terms of a student’s positioning regarding his/her academic studies – for example, the difference between PE (3.04), the highest score, and L_1_ (2.95), the lowest score, is only 0.09. This profile is predominated by a student’s emphasis on positive emotions. The values of L_1_, L_2_, MTL, PIL, and PE are somewhat ‘smaller’ than the values of variables for the other three clusters.

#### Cluster 3 = ‘An Intrinsic Motivation Profile’

Students for this profile place emphasis foremost on personal interest and an inner desire to achieve their learning. The students’ positioning, in this case, is governed by personal interest, which is then followed by their current best practice. When compared to the other three clusters, this cluster as a collective whole is ‘higher’ in values. The difference between PI (4.28) and PE (3.67) is 0.61, which again is higher than the difference between the top value and the bottom value of the other three clusters. Interestingly, unlike the other three clusters, this cluster shows the closeness of four variables – PE, in this case, is somewhat ‘separated’ from PI, L_1_, MTL, and L_2_ (e.g., the difference between L_2_ and PE is 0.41).

#### Cluster 2 = ‘Current Best Practice + Interest Profile’

Students for this profile are realistic in their judgment and perception of best practice. Importantly, this profile emphasizes a student’s realistic measure of his/her ability and, likewise, is governed by PI. What is interesting too, however, is that both L_1_ and L_2_ are consonant with each other in terms of values [i.e., difference in scores between L_1_ (3.57) and L_2_ (3.50) is 0.07]. PI, PE, and MTL, in contrast, are ‘grouped’ together given their distribution of scores, which are similar to each other – for example, the difference between PI (3.24) and MTL (3.14) is 0.10. The difference between L_2_ and PI, 0.26, in this case is larger than 0.10.

#### Cluster 1 = ‘Current Best Practice + Motivation Profile’

Students for this profile, like that of Cluster 2, are realistic in their judgment and perception of best practice. This profile emphasizes a student’s realistic measure of his/her ability and, likewise, is governed by MTL. What is interesting too, however, is that both L_1_ and L_2_ are consonant with each other in terms of values [i.e., difference in scores between L_1_ (3.92) and L_2_ (3.79) is 0.13]. MTL, PI, and PE, in contrast, are ‘grouped’ together given their distribution of scores, which are similar to each other – for example, the difference between MTL (3.56) and PE (3.49) is 0.07. The difference between L_2_ and MTL, 0.23, in this case is larger than 0.07.

## Discussion

The present study is the first, or one of the very few, that sought to explore the topic of ‘profiling.’ Academic profiling is advantageous and may, specifically, inform an educator of how and what a student is thinking at the onset of and/or during the course of his/her learning experience. As a point of reiteration, we proposed a theoretical concept, which we termed as a state of consonance and disconsonance of best practice. A state of consonance considers a close proximity between the two levels of best practice, L_1_ and L_2_, and other comparable psychological variables. Moreover, a state of consonance would depict a closeness or grouping between comparable variables (e.g., L_2_ and intrinsic motivation) whereas, in contrast, a state of disconsonance connotes misalignment or a separation between contrasting variables (e.g., L_2_ and anxiety). As a point of summation, evidence of consonance and/or disconsonance of educational and psychological variables would, to some extent, explain, account, and depict a student’s academic profile.

Our focus of inquiry into the proposition of a state of consonance of best practice, coinciding with an earlier study (Phan et al., 2018a) is insightful, providing potential information into the comparable and comparative profiles of students’ motivational patterns, philosophical beliefs, expectations, etc. As we discuss in this section of the article, the results that we have obtained make both theoretical and empirical contributions, detailing the potency, relevance, and applicability of the consonance-disconsonance framework. A state of consonance of best practice is desirable as it reflects a closeness in association between current best practice, optimal best practice, and other positive-related constructs. A state of disconsonance of best practice, in contrast, is detrimental and may reflect a perceived sense of helplessness and pessimism, as well as a high level of overconfidence. This consideration into the effectiveness of a state of consonance of best practice, aside from practicality, has potential research development for advancement, which we explore in the latter section of this article.

### Theoretical Contribution: The Importance of Student Profiles

University learning is a relatively complex affair as it often entails, for many students, a balance between part-time work and full-time studies (or full-time work and part-time studies). Aside from this personal commitment, students may also have to concurrently enroll in different subject disciplines at any moment in time – for example, a student may have to enroll in an Educational Psychology unit, a Mathematics Education unit, an Asian Philosophy and Cultural Studies unit, etc. Time constraint, domain-specific interest (e.g., a student prefers to study and learn about Asian Philosophy and Cultural Studies unit and not, say, Mathematics Education), and the nature of subject content (Becher, 1987, 1989) may consequently result in different learning experiences and successes and failures. It is not always possible, from this mentioning, for a student to remain ‘optimal’ across all different subject areas.

To seek understanding into university students’ motivational states and learning experiences, a recent inquiry was made, which delved into a concept termed as profiling (Phan et al., 2018a). Our own emphasis in this matter relates to a proposition, similar to that of profiling, that we refer to as a state of consonance and a state of disconsonance of best practice. Using a non-experimental methodological approach, we obtained interesting evidence from university students’ responses that showcased four comparable profiles: a *Balanced Profile*, an *Intrinsic Motivation Profile*, a *Current Best Practice* + *Interest Profile*, and a *Current Best Practice* + *Motivation Profile*. Each profile or cluster, from our analysis, consisted of a number of educational and psychological variables that shared similar characteristics. As shown in Figures 5, 6 as well as Table 6, there is proximity between L_1_ and L_2_ for the four clusters – in this analysis, difference in scores between the two levels of best practice (i.e., L_1_ – L_2_) are 0.13 (Current Best Practice + Motivation Cluster), 0.07 (Current Best Practice + Interest Cluster), 0.10 (Intrinsic Motivation Cluster), and −0.07 (Balanced Cluster). The four differences ranging from 0.07 to 0.13 are relatively minute and, hence, this testament reflects the closeness of L_1_ and L_2_.

Aside from the proximity between the two levels of best practice, we note that both L_1_ and L_2_ also closely associated with the other three variables. For example, from Figure 5 and Table 6, it is interesting to note that the difference is 0.23 between L_2_ and MTL (i.e., between the red bar and the green bar) for the Current Best Practice + Motivation Cluster, 0.26 between L_2_ and PI (i.e., between the red bar and the orange bar) for the Current Best Practice + Interest Cluster, 0.09 between L_1_ and MTL (i.e., between the blue bar and the green bar) for the Intrinsic Motivation Cluster, and 0.02 between L_2_ and PI (i.e., between the red bar and the orange bar) for the Balanced Cluster. The minute values in differences for the four clusters support our emphasis of the consonance of best practice. Importantly, of course, aside from affirming our proposition of consonance of best practice, the four clusters reflect a collective emphasis – namely, a profile of intrinsic motivation and positive emotions for current and optimal best practice.

Although not included, and a possible inquiry for consideration, we speculate that contrasting educational and/or psychological variables would not cluster together – and hence, testament of a state of disconsonance. A state of consonance, in contrast, would indicate favorable and positive profiles for promotion and development. Indeed, from the preceding section, we contend that there are four comparable profiles, which could encourage learning and promote enjoyment and interest (e.g., an Intrinsic Motivation Profile). Moreover, what is of significance from our research inquiry is that it is plausible to categorize students into different groupings or profiles. Each grouping or profile depicts a suite of characteristics and qualities that a student would attest to. This testament of comparable groupings or profiles, we contend, is similar to an earlier study (Phan et al., 2018a), which also showed four distinct profiles (e.g., the Exceptional Profile *versus* the Pessimistic Profile).

**FIGURE 7:**
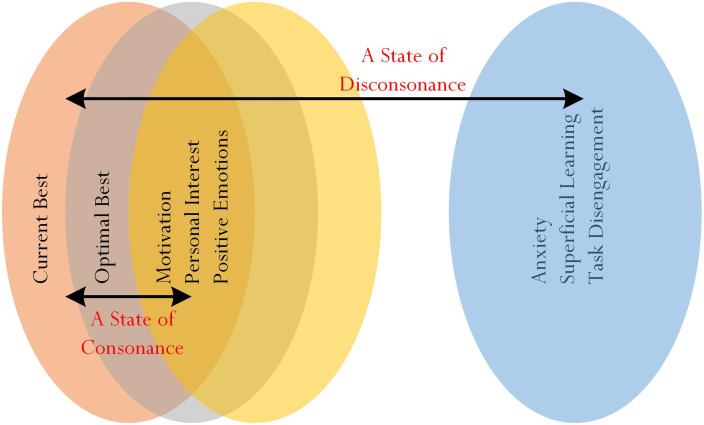
A focus on disconsonance for consideration.

In summary, a specific profile may be evident from a person’s testament of his/her experience of a state of consonance. Speculatively, in this sense, we theorize that a specific profile could associate with performance of different types of adaptive outcomes. For example, from the preceding sections, we contend that a student exhibiting the Intrinsic Motivation Profile would likely attest to his/her inclination towards mastery and engagement in deep, meaningful learning. This consideration, in this analysis, may reflect a state of consonance or a close proximity between the Intrinsic Motivation Profile and mastery and engagement in deep, meaningful learning (e.g., see Figure 7 for guidance). By the same token, however, we would expect to find a state of disconsonance between the Intrinsic Motivation Profile and a student’s disinterest in a subject matter. Indeed, we theorize that profiling may help to categorize a person’s behaviors, thought patterns, and motivational states into distinct clusters, which then could predict his/her future performance in a subject matter.

### Practical Contributions for Consideration

Significantly, aside from theoretical and empirical contributions, the nature of profiling also has a number of potential educational and non-educational implications for consideration. It would be advantageous at the national level, for instance, to collect data and gather information into the academic profiles of students as they enter primary school, secondary school, or university. This national dataset is valuable in terms of assisting institutions with their policy and curriculum development, course offerings, expectations, the allocation of financial resources, etc. For example, a cohort of first-year students who lack the Intrinsic Motivation Profile may require educators to consider pedagogical strategies, programs, on-campus activities, etc., which could help encourage and promote the adoption of the Intrinsic Motivation Profile. In a similar vein, perpetual datasets available may allow institutions, stakeholders, educators, etc. to explore and identify specific longitudinal trajectories of differing profiles (e.g., 65% of first-year students exhibited the Balanced Profile over the past 3 years).

At the institution level, a focus on profiling is effective for its potential impact on the creation and promotion of a positive on-campus climate and culture for learning. The study of positive psychology (Seligman et al., 2009; Seligman, 2010; Kern et al., 2019), recently emerged as an important focus for research development, is valuable, helping to facilitate the proactivity of human agency. For example, one application of the tenets of positive psychology is closely aligned with the promotion of positive emotions, such as a person’s state of happiness (e.g., Chan, 2013; Hasnain et al., 2014; Donaldson et al., 2015; Tabbodi et al., 2015). Feeling good about oneself, academically and/or non-academically, likewise, also reflects the significance of positive psychology. A positive learning climate and/or a proactive on-campus culture (e.g., availability of opportunities for daily, weekly, and/or monthly extracurricular activities), in this sense, would instill and foster positivity. The question then, from this consideration, is whether and to what extent positive academic profiles would help cultivate and sustain a proactive on-campus and culture for learning. Evidence of an institution’s attempt to promote and cultivate positive academic profiles, via means of policies, course offerings, etc. would, in this case, instill perception of care, understanding, and compassion.

At the individual level, we contend that the study of profiling is noteworthy for its diagnostic possibility – that is, knowing about a student’s specific profile may assist educators to develop personal measures, tools, etc. that could counter or encourage the continuation of such profile. An undesirable profile, which consists of negative characteristics (e.g., a student’s tendency towards pessimistic thoughts), for example, would require some form of remedy and prevention. What can a student do to negate such tendency and/or adoption of an undesirable profile? Research in the early 1980s, interestingly, focused on the use verbal discourse (e.g., encouraging feedback) to encourage ad motivate positivity (e.g., Schunk, 1982, 1983, 1984). More recently, however, researchers have focused on usage of pedagogical strategies that could facilitate effective learning and deep, meaningful understanding of subject matters (Ngu and Yeung, 2012, 2013; Walkington, 2013; Ngu et al., 2014, 2015). At the same time, however, we strongly believe that desirable profiles for learning (e.g., the Intrinsic Motivation Profile) could operate as sources of positive psychology (Seligman et al., 2009; Seligman, 2010; Kern et al., 2019) and motivation for learning. In this analysis, desirable academic profile (e.g., the Intrinsic Motivation Profile or the Exceptional Profile: Phan et al., 2018a) are perceived as being positive and motivational, unlike undesirable profiles, which are negative and detrimental.

### Research Caveats for Future Research Development

Aside from theoretical and practical contributions, our research investigation has also identified a number of caveats that may assist in the continuation of this line of inquiry into the study of optimal best. Foremost, from the preceding sections, our focus of inquiry into profiling and the importance of a state of consonance of best practice was ‘positive,’ and consisted of the deliberate choosing of psychological variables that are positive, in nature. As such, it was somewhat difficult for us to establish disparate and/or contrasting patterns of both L_1_ and L_2_ with other variables (e.g., a state of disconsonance between L_1_ and, say, anxiety). An earlier study, in contrast, was able to identify different patterns of both L_1_ and L_2_ (e.g., low L_1_ and high L_2_) (Phan et al., 2018a). In this sense, an inspection of our conceptualization and subsequent results suggests one notable caveat, which namely consisted of our exclusion of maladaptive outcomes and/or negative life experiences.

For clarity and holistically, it would be of interest in future research to explore a state of disconsonance and how this state could potentially explain and/or account for different types of negative life experiences. A state of disconsonance of best practice (e.g., Figure 3) would, in this case, involve examination of educational and/or psychological variables that are non-compatible or dissimilar in terms of their characteristics and qualities. From existing research, we know that personal self-efficacy for academic learning (Bandura, 1986, 1997) is inversely associated with a state of apprehension or anxiety (Pajares and Kranzler, 1995; Jain and Dowson, 2009; Villavicencio and Bernardo, 2016). Anxiety, superficial learning, and/or task disengagement, likewise, are variables that share similar negative characteristics with each other, and may closely associate with different types of detrimental outcomes (e.g., underachievement in a subject matter). A state of disconsonance, in this case, would indicate a distance proximity and/or a misalignment between a high level of L_1_ and a high level of L_2_ and anxiety, superficial learning, and/or task disengagement.

Our recommendation for research development, as shown in Figure 7, depicts a state of disconsonance between different types of ‘non-related’ or ‘misaligned’ variables within a system of change (e.g., L_2_ and superficial learning). Importantly, from this proposition, we speculate two distinct groupings: one positive grouping (e.g., Group 1), consisting of L_1_, L_2_, MTL, PIL, and PE, and one negative grouping (e.g., Group 2), consisting of, say, anxiety, superficial learning, and task disengagement (Note: other variables may include pessimism, a perceived sense of helplessness, and confusion). For investigation, we posit that *low levels* of L_1_, L_2_, MTL, PIL, and PE (i.e., Group 1) would closely associate with *high levels* of anxiety, task disengagement, and other maladaptive processes and/or outcomes (i.e., Group 2) (i.e., a state of consonance). In contrast, however, we consider a state of disconsonance as being the relationship (or the farness in proximity) between *high levels* of L_1_, L_2_, MTL, PIL, and PE and *high levels* of anxiety, task disengagement, and other maladaptive processes and/or outcomes. On this basis, it would be of interest, theoretically, for researchers to explore and validate different states of disconsonance for different educational and psychological variables (e.g., a high level of L_2_ and a high level of anxiety *versus* a high level of L_2_ and a low level of anxiety). Testament of a state of disconsonance may, in this case, assist educators to localize psychosocial factors and/or psychological variables, which could negate students’ confidence, beliefs, and perceptions of their abilities.

Another focus of inquiry that we recommend relates to a student’s academic profile that may situate within the context of his/her sociocultural backgrounds. There is acknowledgment that historical upbringing and sociocultural factors, encompassing collective values, customary practices, philosophical beliefs, and expectations may influence students’ motivational states and learning experiences. Taiwanese, in general, strongly align themselves with collective thinking (Triandis et al., 1988; Markus and Kitayama, 1991) and the notion of *filial piety* (Chow and Chu, 2007; Hui et al., 2011; Chen, 2016), which emphasizes dutiful behaviors, obligation to parents and elders, respect, and the importance in family values. Taiwanese students work hard academically so that their achievements and successes are celebrated and shared by immediate and distant family members. Continuing mediocre performances and/or failures, in contrast, would bring shame and dishonor to the family, resulting in the perception that one has not been dutiful. Given this understanding, we query whether established patterns of consonance of best practice (e.g., Figure 5) in our study could have been different (or similar) for students of other sociocultural backgrounds? This question places emphasis on theoretical understanding and self-awareness of different cultures.

Finally, referring back to our earlier discussion, the sample used for this study was convenient in nature and consequently, on this basis, evidence obtained from our analyses is somewhat limited in terms of generalization. Indeed, rather limited in nature, our study involved the use of a convenient sample, which was biased and limited the subsequent analyses and findings for discussion. Ideally, in this sense, we would have preferred to use a larger dataset that could, likewise, depict multiple ‘systems’ within the context of education – for example: public and private universities that are located in metropolitan and regional cities. In Taiwan, there two types of university: public *versus* private. Public universities are seen and perceived as being more prestigious, making it extremely competitive in terms of entry into both undergraduate and postgraduate degree programs. In general, families prefer their children to go to public universities as this attendance would bring prestige, pride, and perceived success. As such, students who attend public universities are perceived as being top-tier, more ‘intelligent,’ academically-minded, and motivated. Second-tier students who are not able to gain entry into public universities instead attend private universities, which are less esteemed and require personal financial funding from families (i.e., families have to fund their children). Logistic limitations, time constraint, and institutions’ unwillingness to take part made it somewhat difficult for us to collect a larger randomized sample. Ideally, of course, it would have been a favorable endeavor for us to use randomized sampling and, on this basis, to be able to generalize our results to the wider population in terms of differing manifestations of learning and motivational profiles. We urge researchers to perhaps engage in multiple-institutional collaborations, which could help address the limitation of our sampling and/or to improve the sample sizes. By all account, it is plausible that the sample used in the present study is unique, cross-culturally, giving rise to the established results. It is also a possibility, of course, that the sample used in our study is comparable with samples found elsewhere, indicating that what we have found is common.

## Conclusion

The present research investigation, overall, enabled us to advance the study of best practice, which in this case inquired into the concept of profiling. Academic profiling may indicate a student’s learning pattern and, possibly, his/her state of motivation. Our results, non-experimentally, provided support for an important concept, which we termed as the consonance-disconsonance of best practice. Consonance of best practice posits that different levels of best practice (e.g., low level of best practice *versus* optimal level of best practice), as well as other positive psychological constructs (e.g., motivation towards learning) would ‘group’ together. Disconsonance of best practice, in contrast, would indicate non-overlapping of contrasting levels of best practice (i.e., low level of best practice *versus* optimal level of best practice). From this testament, we were able to identify four comparable profiles that emphasize the importance of consonance and disconsonance of best practice: Balanced Profile, an Intrinsic Motivation Profile, a Current Best Practice + Interest Profile, and a Current Best Practice + Motivation Profile. This seminal evidence is insightful and may, we hope, provide empirical grounding for further development into the topic of profiling.

## Data Availability Statement

The data analyzed in this study is subject to the following licenses/restrictions: the universities involved do not allow the sharing of this dataset. Requests to access these datasets should be directed to HP, hpha7292@yahoo.com.

## Ethics Statement

The studies involving human participants were reviewed and approved by University of New England Research Ethics Committee. We verbally sought permission and informed any participant who did not wish to participate to let us know at the onset. Written informed consent for participation was not required for this study in accordance with the national legislation and the institutional requirements.

## Author Contributions

HP was responsible for data collection, articulation of conceptualization, and write-up of the manuscript. BN was responsible for articulation of conceptualization, data analyses, and write-up of the manuscript. Both authors contributed to the article and approved the submitted version.

## Conflict of Interest

The authors declare that the research was conducted in the absence of any commercial or financial relationships that could be construed as a potential conflict of interest.
